# 

**Published:** 1831-06

**Authors:** 


### MONTHLY LIST OF MEDICAL BOOKS.

[*Medical Works cannot be entered on this List unless a copy be sent for the purpose, the titles of Books having frequently been sent to us as published which have not been published for weeks, or even months, after.*]

Distinction without Separation: in a Letter to the President of the College of Surgeons, on the present State of the Profession. By JOSEPH HENRY GREEN, F.R.S. F.G.S., Professor of Anatomy to the Royal Academy, &c.—Pp. 47. Hurst and Co. London, 1831.

Observations on Distortions of the Spine, with a few Remarks on Deformities of the Legs. By LIONEL J. BEALE, Member of the College of Surgeons, and Author of a "Treatise on Deformities."—8vo. pp. 102. Wilson, London.

The History of Medicine, Surgery, and Anatomy, from the Creation of the World to the Commencement of the Nineteenth Century. By WM. HAMILTON, M.D. Vol. II.—8vo. Colburn, London.

Essai sur la Raison et la Folie: Thèse Inaugurale, presentée et soutenue à la Faculté de Médecine de Paris. Par J. BYRON BRADLEY, de l'Isle de Jamaïque, M.D. B.L.—Paris.

### NOTICES.

*Communications have been received from* DR. BOW, MR. MITCHELL, &c.

### INDEX

TO THE TENTH VOLUME OF THE NEW SERIES

OF

THE LONDON MEDICAL AND PHYSICAL JOURNAL.

---

PAGE

ABDOMEN, pressure of, to expedite delivery . . 276
Abernethy, death of Mr. . 569
Abstinence, on dangers of prolonged, in various diseases, 296, 392
———, influence of, upon the blood and muscles . 297
——————————— diseases of the heart often injurious 300
Acupuncturation, remarks on . 562
Air in veins of the neck; fatal consequences of, during operations 122
Alimentary canal, difficulty of ascertaining marks of inflammation in, after death . 239
——————— hyperæmia of 240
——————— anæmia of . 241
——————— hypertrophy of, ib.
——————— softening of 245
——————— ulceration of 249
——————— perforations of, 250
——————— changes of capacity in . . 252
——————— on abstinence in diseases of . . 395
Amaurosis from irritation of fifth pair of nerves . . 73
———, cases of . 365
Amputation, propriety of immediate, after receipt of violent injuries . . 125
——— of the leg, cases of 120
——— of both legs after mortification . . 320
Anatomy, M. Andral on Morbid 234
——— Mr. Paxton's Introduction to . . 269
Aneurism of the external iliac; ligature applied to the common iliac . . 78
——— of subclavian and carotid, cured by ligature . 144
———, case of axillary; subclavian tied . . 507
Anus, constriction of, relieved by belladonna . . 364

PAGE

Anus, remarks on diseases of . 138
Apothecaries' Company; rules laid down for lecturers . 471
Arsenic given in large doses . 86
Arteries, case of disease of femoral and iliac, with gangrene and paralysis . . 131

Barry, Dr. Reply to Mr. Fraser, 99, 475
Belladonna, used in spasmodic strictures of urethra . 283
——— in hooping cough . 171
——— in hydrophobia . 275
———, use of, in constriction of anus . . 364
——— a preventive of scarlet fever 361
Blushing, on the phenomenon of 453
Body, influence of diseases of, on the mind . . 278
Bones, on the reunion of fractured 423
Botany, Introduction to a Natural System of . . 45
———, Introduction to Medical 450
Brain, softening of, with paralysis 321
———, loss of large portion of, from a wound: recovery . 359
———, on abstinence in diseases of 396
———, affection of, relieved by food 397
Breast, removal of, for scirrhus 513
Bronchia, foreign body remaining ten years in, before death . 85
Broussais, observations on his doctrines . . 15
Burnett, Mr. G. T., appointed professor of botany to King's College, 473
Burns, tincture of benzoin recommended in . . 85
Butchers generally healthy . 518

Cancer of rectum, excision of . 275
Cantharides, saturated infusion of, in vinegar, as a counter-irritant 69
———, poisoning by . 362
———————, followed by expulsion of mucous membrane of the œsophagus . 77

PAGE
Carditis, cases of . . 293
Charcoal, useful in poisoning with Hydr. Mur. . 169
Chessher, Mr. Robt. biography of 374
Children, serious cerebral symptoms often produced in, by low diet 398
Chorea . . . 418
———, utility of subcarbonate of iron in . . 420
Cicatrix, removal of an extensive, after burns . . 43
Ciliary ligament, growth from 460
Climate, influence of tropical, in consumption . 199
Colica constipata, cured by inflation, 558
College of Surgeons, notice from, respecting the library and publication of "Transactions" . 471
Colon, on spasmodic stricture of 161
Combustion, remarks on spontaneous, of the body . 515
Consumption, on inhalation of various medicines in . 63
——— and scrofula, connexion between . 105
Convulsion, peculiar species of, in twins . . 23
Cornea, on cauterization of, in diseases of the eye, with dilated pupil . . 181
Cretinism . . 347

Delirium tremens, cases of . 38
Diseases, connexion between, with the rock formations of a country 87
"Distinction without Separation," a letter addressed by Mr. Green to the President of the College of Surgeons; review of 534

Œsophagus, case of foreign body in, 272
Electric and nervous fluids, identity of, attempted to be proved by experiments . 454
Emphysema uteri, case of . 391
——— formed by a combustible gas . . 515
Empyema: Paracentesis thoracis performed with success . 512
Endermic applications of medicines, 171
Epiglottis, danger of suffocation, from falling of . 266
Epistaxis, fatal case of . 112
Ergot of rye, on the use of, in 720 cases . . 463
Esophagus, mucous membrane of, expelled, after poisoning by cantharides . . 77
Exhalations, innocuous nature of putrid 518

PAGE
Eye, extirpation of a cancerous 83
—, mode of extracting foreign bodies from, in some parts of Germany . . 561

Fever, origin of Gibraltar, of 1828, 286
Fistula in ano, connexion between, and phthisis . 110
Fits relieved by moxa . 44
Flax workers, their health injured by their trade . 523
Fœtus, physiology of the 427, 543
Fracture, case of ununited, of the thigh, cured . . 126
——— of the leg, with amputation, 41
———, followed by tetanus, 42
Fraser, Mr., reply to Dr. Barry on the Gibraltar Epidemic, 31, 202, 304, 399

Gallic acid, the active ingredient of "Ruspini's styptic" . 188
Gas, general emphysema formed by combustible . 514
Gibraltar epidemic of 1828, remarks on 31, 92, 99, 202, 304, 399, 475
Gibraltar fever of 1828, origin of 286
Glossitis, cases of idiopathic . 457
Goitre . . 347
Gratiola (hedge hyssop), use of, in delirium tremens . 273
Guthrie, G. J. F.R.S., his Introductory Lecture at the College of Surgeons . 464

Heart, abstinence often injurious in diseases of . . 300
——, cases of organic disease of, 379, 497
——, average weight of . 380
——, case of ossification of 456
——, diseases of, frequently mistaken for consumption . 385
——, bleeding to be used with caution in organic diseases of 388
——, distinction between organic diseases and functional derangements of . . 383
——, various morbid conditions of, 329
Hernial sac, inflammation of, resembling strangulation . 514
Hernia, omentum tied and removed in a case of strangulated . 510
———, on the taxis in strangulated, 369
———, propriety of free purging after operation for strangulated, doubted . . 185
——— produced occasionally by the patient's straining to make water, when labouring under stricture . . 152
Hooping cough, belladonna in 171

PAGE
Hydrocephalus, caution respecting tapping in . . 98
Hydrophobia, belladonna used in 275
Hysteria connected with spinal irritation . . 4
Hysteritis, case of . . 104

Iliac artery, ligature applied to the common . . 78
Infant, sudden death of an . 494
Infants, mortality of . 373
Inhalation of various medicines in consumption . . 62
Intestine, case of rupture of . 264
———, internal strangulation of 224
———, various causes producing internal strangulation of . 254
Intestines, causes of redness of mucous membrane of . 237
————, collection of fæces in, producing symptoms of internal strangulation . . 169
————, on diseases of, below diaphragm . . 236
————, redness of, not always indicative of inflammation . 237
————, tympanitis of, relieved by introducing a tube . 456
————, various lesions in 240
————, increase of air in, from disease . . 256
Iris, absorption of . 460
Ischuria renalis, case of . 28

Jaundice without disease of liver 343
Joints, cases of purulent deposition in, after injuries . 230

Lagophthalmia, proposed operation for . . . 561
Laryngitis, tracheotomy often necessary in . . 159
Leg, cases of amputation of . 120
—— amputation of, at knee-joint 180
Lithoplatomy, on . 225
Lithotomy, on a new mode of performing operation of . ib.
Lithotrity, Baron Heurteloup's cases of . . 441
———, Civiale's report of cases of, 562
Liver, morbid appearances of 341
——, physiology of . 432
Long, Mr. St. John, his liniment 184
Lungs, abstinence often injurious, in diseases of . . 392
———, modes of ascertaining capacity of diseased . 272

Magennis, Dr. death of . 88
Malsters, their health injured by their trade . . 522

PAGE
Malaria in Italy . 353
Manufactures, influence of, upon health . . 517
Masons usually short-lived . 524
Medical degrees not granted at the London University or King's College . . 277
——— witnesses cannot claim remuneration . . ib.
——— practitioners not liable to serve on juries . 472
Medicines, new mode of preparing with sugar . . 183
Melanosis . . . 257
Menorrhagia, utility of savin in 361
Mercury, singular result from external use of muriate of . 463
Midwifery Report . 103
Millers, their health injured by their trade . . 522
Mouth, gangrene of . 327
Moxa successfully used in a case of "fits" . . 44

Nannari, a substitute for sarsaparilla, 189
Naval surgeons admitted to the King's levees . 374
Nerve, hypertrophy of pneumogastric . . 244
Nerves, diseases of, frequently not to be detected by dissection 344
Nervous and electric fluids, identity of, attempted to be proved by experiment . 454
Neuralgia cured by spontaneous ptyalism . . 273

Paracentesis thoracis performed with success by Mr. Mayo . 512
Patella, on fracture of . 567
Paxton, Mr., notice of his "Introduction to the Study of Human Anatomy" . 269
Pellagra . 351
———, pathology of . 168
Perforations occurring in stomach and intestines . . 250
Pharmacopœia Universalis 528
Phlebitis cured by compression 37
Phlegmasia dolens, and swelling of limbs after fever, identity of 136
Phthisis, on inhalation of various medicines in . 63
——— and scrofula, connexion between . . 105
———, danger of repressing symptoms of scrofula in 107
———, influence of tropical climates in . . 199
———, connexion between, and fistula in ano . . 110

PAGE

Physicians, necessity of some reform in the constitution of the College of . . 535
Piles, on the best mode of applying ligature to . . 185
Placental soufflet, remarks on the 258
Poisoning with Hydr. Mur., charcoal useful in . 169
Pregnancy, use of the stethoscope in detecting . . 258
Pressure of the abdomen in obstetrics . . 276
Psoriasis successfully treated . 267

Ramadge, Dr., expelled from the London Medical Society . 568
Rectum, excision of cancer of 275
———, on diseases of . 138
———, on stricture of . ib.
———, vascular tumors of; excision or ligature? . 142
———, on structure of mucous membrane of . 143
———, ulcerative disease of, removed by excision, by Mr. Mayo 319, 510
Respiratory apparatus, lesions of 339
Ribs, case of fractured, followed by empyema: cure by paracentesis thoracis . . 512
"Ruspini's styptic," composition of, 187

Scald, case of severe, treated by nitrate of silver . 564
Scalds, tincture of benzoin recommended in . . 85
Scarlet fever prevented by belladonna . . 361
Scrofula, danger of repressing symptoms of, in phthisis . 107
——— and consumption, connexion between . 105
Secale cornutum, on the use of, in 720 cases . . 463
Spinal cord, functional disorders of 1
———, irritation of, causing hunger . . 1
Spleen, Physiology of the . 432
Stethoscope, use of, in detecting pregnancy . . 258
Stomach, abundant secretion of fluid in . . 255
———, causes of redness of its mucous membrane . 237
———, hernia of . 76
———, on diseases of . 236
———, connexion between cramp of, with disease of the brain . 2
———, redness of, produced by digestion, continues after death 236
Stone in the bladder, Mr. Mayo's clinical lecture on . 33

PAGE

Straw-bonnet makers injured by their trade . . 521
Subclavian artery, mode of tying 145
Suffocation, danger of, from falling down of the epiglottis, in a case of attempted suicide . 266
Surgeons, members of the College of, cannot send medicines to their patients, unless they belong to the Apothecaries' Company . 374
———, observations on the want of power of the College of, to protect their members . 535
Syncope not a constant symptom in heart diseases . 382
Syphilis cured by mercurial pediluvia, 460
——— cinnabar fumigations, 461

Tailors, diseases common among 520
Tanners generally healthy . 521
——— exempt from consumption ib.
Tapping in hydrocephalus, caution respecting . . 99
Testicle, voluntary power of drawing up, so as to imitate hernia 277
Tetanus, case of trismatic, cured by tobacco injections . 114
——— cured by injecting opium into the veins . 560
——— injections of stramonium ib.
———, cases of traumatic . 322
Thigh, case of ununited fracture of, cured . . 126
Tic douloureux cured by extracting a tooth . 195
Tonsils, morbid alterations of . 327
Tracheotomy, frequent propriety of, in bronchitis . 159
Tumor weighing fifty-six pounds, removal of . . 414
Tympanitis of the intestines relieved by introducing a tube 456

Unio pictonum, fertility of . 86
Urethra, treatment of strictures of, 153
———, strictures of . 148
———, recurrence of, not always to be prevented . 154
———, division of, in perinæo . . 157
———, their ordinary situation . . 149
———, changes in mucous membrane during . ib.
——— not arising from injections, if judiciously employed, 150
———, on irritable . 151
———, organic disease of testicle from . ib.
———, use of belladonna in spasmodic strictures of . 283

PAGE
Urethra, strictures of, sometimes lead to herniæ, from straining in making water . 152
Urine, cases of congenital incontinence of . . 554
Uterus, prolapsus and rupture of, during labour: recovery . 461
———, case of emphysema of the 391
———, on gaseous tumors of, resembling pregnancy . 360

Vaccination, Mr. Marshall's popular summary of . 377
Vaccine virus, cow's milk said to communicate to variolous virus the properties of . 76
Variola, calamine used to prevent the pits of confluent . 560
Vein, inflammation of saphena, with symptoms of ague . 135
Veins, on the dangerous and fatal effects from admission of air into, during operations in the neck . . 175
Vision, on double . 72
Vomiting, on the mechanism of 270

Wool, important fact connected with manufacture of . 526

Yellow fever on board the Sybille, remarks on Dr. M'Kinnal's report . . 18

---

### CRITICAL ANALYSES.

An Introduction to the Natural System of Botany. By J. LIMDLEY, F.R.S. 45
Cases illustrative of the Efficacy of inhaling various Medicines in Pulmonary Consumption, &c. By Sir C. SCUDAMORE, M.D. F.R.S. . 62
A Practical Treatise on Diseases of the Eye. By W. MACKENZIE, Esq. 70
Dublin Hospital Reports and Communications, &c. Vol. V. . 131, 258
Surgical Observations on the Diseases of the Mucous Canals. By G. MACILWAIN, Esq. . . . . . . 148
Practical Remarks on the Discrimination and successful Treatment of Spasmodic Stricture in the Colon. By JOHN HOWSHIP, Esq. . 161
A Treatise on Pathological Anatomy, by G. ANDRAL. Translated from the French, by Drs. TOWNSEND and WEST. Vol. II. . 234, 326
An Introduction to the Study of Human Anatomy. By J. PAXTON, Esq. 269
Change of Air, or the Pursuit of Health, &c. By J. JOHNSON, M.D. &c. 345
The Physiology of the Fœtus, Liver, and Spleen. By GEORGE CALVERT HOLLAND, M.D. &c. . . . . . 427, 543
Cases of Lithotrity, &c. By Baron HEURTELOUP, D.M.P. . 441
An Introduction to Medical Botany, &c. By THOMAS CASTLES, F.L.S. 450
The Effects of the principal Arts, Trades, and Professions, &c. on Health and Longevity. By C. T. THACKRAH . . . . 516
The Pharmacopœia Universalis, by A. J. L. JOURDAN, M.D. of Paris. Edited by J. RENNIE, A.M. A.L.S. . . . . 528
Distinction without Separation: in a Letter to the President of the College of Surgeons, on the present State of the Profession. By J. H. GREEN, F.R.S. 534

---

COLLECTANEA . 76, 168, 270, 359, 453, 554
INTELLIGENCE 87, 184, 277, 374, 471, 568
METEOROLOGICAL REGISTER . 90, 186, 282, 378 474, 570

### NAMES OF AUTHORS,

*Of whose Works, Observations, &c. a more or less detailed Account is given in this Volume.*

AGE

ANDRAL, Professor, Treatise on Pathological Anatomy . 234, 326
ARMOUR, Dr. on the use of Secale Cornutum . . 462
ASHBURNER, Dr. on Nannari, or East-India Sarsaparilla . . 189
BALLY, M. case of general Emphysema formed by Combustible Gas . 514
BANKS, Dr. TATAM, on Acupuncturation . . . 562
BARLOW, Mr. on the fatal Effects arising from the Entrance of Air into the Veins of the Neck during Operations . . . 175
BARRY, Dr. DAVID, case of Tic Douloureux . . 195
————, on the Gibraltar Epidemic . . . 91
————, Reply to Mr. Fraser . . . 99, 476
BERAL, M. on Preparation of Medicines with Sugar . . 183
BIRD, J. Esq. case of Ischuria Renalis . . . 28
BOILEAU, M. case of Foreign Body in the Œsophagus . . 272
BUCHANAN, Dr. M. S. on Lithoplatomy . . . 225
CARESI, Dr. case of Facial Neuralgia cured by spontaneous Ptyalism . 273
CARTER, JESSE, M.D. cases of Mania a Potu . . 38
CASTLE, THOMAS, F.L.S. review of his Introduction to Medical Botany, &c. 450
————, notice of his Manual of Surgery . 184
CAUMONT, M. DE, on the Connexion of Diseases with the Rock Formations of a Country . . . . . . 87
CIVIALE, Dr. Report of Lithotritic Cases . . 562
COLLES, Professor, on Diseases of the Anus and Rectum . . 138
CRAMPTON, PHILIP, M.D. F.R.S. case of Aneurism of the External Iliac: ligature applied . . . . . 78
DARKIN, Dr. on large Doses of Arsenic . . . . 86
DAVID, Dr. on the Identity of the Nervous and Electric Fluids . 454
DUPUYTREN, M. case in which a Foreign Body remained ten years in the Bronchia before causing Death . . . 85
————, cases in which Fatal Results were produced by the Entrance of Air into the Veins . . . . 122
ELLIOTSON, Dr. on St. Vitus's Dance . . . . . 418
FAHNESTOCK, W. M. M.D. on Compound Tincture of Benzoin as a Remedy for Scalds and Burns . . . . . 85
FRASER, Mr. HUGH, Reply to Dr. BARRY . 31, 202, 304, 399
GAITSKELL, Dr. account of the Sudden Death of an Infant . 494
GUYON, M. on Gibraltar Fever . . . . 286
GEORGE, Mr. on use of Calamine to prevent the Pits of Confluent Smallpox 560
GERHARD, Dr. on the Endermic Use of Medicines . . 171
GRAVES, Dr. Clinical Report of Meath Hospital . . 131
GREEN, J. H. F.R.S. &c. analysis of his Letter to the President of the Royal College of Surgeons, entitled "Distinction without Separation" 534
GRIFFIN, Dr. on Functional Disorders of the Spinal Cord 1
GRIFFIN, D. Esq. on Functional Disorders of the Spinal Cord . ib.
GUERSENT, M. case of Softening of the Brain, with Paralysis . 321
GUTHRIE, G. J. F.R.S. Introductory Lecture delivered at the College of Surgeons . . . . . . 464
———— cases of Amputation . . . 119
HALFORD, Sir HENRY, on the Influence of Diseases of the Body on the Mind, 278
HALL, Dr. MARSHALL, on the Mechanism of Vomiting 270
————, cases of Convulsions in Twins . . 23
HART, Mr. case of Ruptured Intestine . . . 264
HENSCHEL, Dr. case of Prolapsus and Rupture of the Uterus during Labour. Recovery . . . . . 461

PAGE
HEURTELOUP, Baron, review of his Cases of Lithotrity . . 441
HOLLAND, G. C. M.D. on the Physiology of the Fœtus, Liver, and Spleen, (analysis) . . . . . 427, 543
HORT, W. P. M.D. case of Poisoning with Hydr. Mur. . 169
HOUSTON, Dr. case of Attempt at Suicide, with Danger of Suffocation from falling down of the Epiglottis . . . . 266
HOUSTON, Mr. on the Mucous Membrane of the Rectum . 143
HOWSHIP, J. Esq. on Spasmodic Stricture of the Colon, &c. . 160
JOHNSON, JAMES, M.D. on Change of Air, or the Pursuit of Health (review) 345
JOURDAN, A. J. L. account of his Pharmacopœia Universalis, &c.; edited by Mr. RENNIE . . . . . 528
JÜNGKEN, Dr. on Operation for Lagophthalmia . . 561
KENNEDY, Dr. E. on the Placental Soufflet . . . 258
KEY, Mr. case in which a Tumor, fifty-six pounds in weight, was removed 414
KING, JOHN, jun. case of Colica Constipata, removed by Inflation . 558
KNOX, J. J. A.M. on Amaurosis . . . . . 365
LARBORDERIE, M. on Belladonna in Constriction of Anus . . 364
LARREY, Baron, on the Propriety of immediate Amputation after severe Gunshot Wounds . . . . . 125
LARUSSAC, Dr. case of Internal Strangulation . . . . 224
LAUTH, M. E. A. on the Phenomenon of Blushing . . 453
LINDLEY, JOHN, F.R.S. L.S. G.S. &c. analysis of his Introduction to the Natural System of Botany . . . . . 45
LINDSLY, H. M.D. case of Extirpation of a Cancerous Eye . 83
LISFRANC, M. on Excision of Cancer of the Rectum . . . 275
LISTON, R. Esq. notice of his Elements of Surgery . . 88
LONG, EDWIN, Esq. on the Use of Belladonna in Spasmodic Strictures 283
LUCCA, Dr. on the Pellagra at Milan . . . 168
MACILWAIN, Mr. GEORGE, on Diseases of Mucous Canals . . 148
MACKENZIE, W. Esq. analysis of his Treatise on Diseases of the Eye 70
MAYO, HERBERT, F.R.S. case of ulcerative Disease of the Rectum removed by Excision . . . . . 319, 510
——————, Amputation of both Legs after Mortification 320
——————, case of Compound Fracture of the Leg; Amputation 41
——————, case of Compound Fracture of the Leg, followed by Tetanus . . . . . . 43
——————, Removal of a Cicatrix after a Burn . ib.
——————, Fits relieved by the Use of Moxa . . 44
——————, case of Strangulated Hernia; Entero-Epiplocele; the Omentum tied and removed . . . . 510
——————, case of Fractured Ribs; Empyema; Paracentesis Thoracis . . . . . . 512
——————, Removal of Scirrhus Breast . . 513
——————, on Stone in the Bladder . . 33
MIDDLEMORE, Mr. on Absorption of the Iris . . . 460
—————— on a Growth from the Ciliary Ligament . ib.
MIGUEL, Dr. on Belladonna in Hooping Cough . . . 171
MITCHELL, C. Esq. cases of Carditis . . . 203
—————— case of Emphysema Uteri . . 391
M'LEOD, G. Esq. on Cure of Hernia by the Taxis . . 369
M'MORRIS, Mr, on Abdominal Pressure in Obstetrics . . 276
MOTT, VALENTINE, M.D. case of Axillary Aneurism . . 507
MUKEBECK, M. on the Use of Gratiola in Delirium Tremens . 273
ORGILL, JOHN, Esq. cases of Glossitis . . . 457
OTTO, J. C. M.D. cases of Congenital Incontinence of Urine . 554
PARRISH, Dr. on the Connexion between external Scrofula and Consumption 105
PAXTON, JAS. Esq. Introduction to Human Anatomy . . 269
PERCY and LAURENT, MM. on the Cure of Tetanus . . 560
PERCY, Dr. case of Traumatic Tetanus . . . 322
PIORRY, P. A. on the Dangers of Abstinence . . 296, 393

PAGE
PORTER, Mr. cases of Aneurism . . . . 144
RECAMIER, M. case of Phlebitis cured by Compression . . 87
RICHARDSON, JAS. Esq. case of Ossification of the Heart . 456
ROBER, Dr. on the Vaccine Virus . . . . . 76
ROUQUARIOL, M. case of Poisoning by Cantharides . . . 77
SANITARIUS, on the Yellow Fever on board the Sybille . . 18
SANSON, M. case of Inflammation of a Hernial Sac, with Symptoms of Strangulation; Operation . . . 514
———— case of Collection of Fæces in the Intestines, producing Symptoms of internal Strangulation . . . 168
SAZYMA, Dr. on the Power of Belladonna to prevent Scarlet Fever . 361
SCUDAMORE, Sir CHAS. M.D. F.R.S. analysis of his Cases illustrative of the Efficacy of various Medicines administered by Inhalation in Pulmonary Consumption . . . . 62
SEERIG, Professor, notice of his Anatomical Demonstrations . . 89
SERRES, M. on the Cauterization of the Cornea in Diseases of the Eye 181
SIEVWRIGHT, F. Esq. Fatal Case of Epistaxis . . 112
SMART, Dr. B. case of Trismatic Tetanus . . . 114
SOMME, Dr. Cure of ununited Fracture of the Thigh . . 127
STEFANO, Dr. case of Wound of Head, with Loss of large Portion of Brain 359
STEPHENSON, J. M.D. F.L.S. notice of his Medical Zoology . 185
STOKES, Dr. Clinical Report of Meath Hospital . . . 131
SYME, Mr. on Fracture of the Patella . . . 567
————, on Reunion of Fractured Bones . . . 423
TAMBONE, Dr. on the Cure of Syphilis by Mercurial Pediluvia . 460
THACKRAH, C. T. on the Effects of Arts, Trades, and Professions on Health and Longevity . . . . . . 516
THOMSON, Dr. A. T. notice of his Introductory Lecture to a Course of Lectures on Medical Jurisprudence . . 281
————————, on the Composition of "Ruspini's Styptic" . 187
TOUZET, Dr. case of Tympanitis of the Intestines, relieved by the Introduction of a Tube . . . . 456
UNGER, Dr. on the Fertility of the Unio Pictonum . . 86
VELPEAU, M. on Amputation of the Leg at the Knee-joint . 180
WADE, R. Esq. cases of Organic Disease of the Heart . 379, 497
WALLACE, J. Esq. on Consumption in Tropical Climates . 198
WALLER, C. Esq. Half-yearly Midwifery Report . . . 103
WEDEKIND, Dr. on the Use of Savin in Menorrhagia . . 361
WEIR, Dr. cases of Purulent Deposition in Joints, after Injuries . 230
WERNECK, Dr. Treatment of Syphilis by Cinnabar Fumigations . 461
WEST, Dr. W. case of Psoriasis, successfully treated . . 267
WILLIAMS, Mr. case of Poisoning by Cantharides . . 362

THE END.

J. AND C. ADLARD, PRINTERS,
BARTHOLOMEW CLOSE.

